# Monocyte gene expression in childhood obesity is associated with obesity and complexity of atherosclerosis in adults

**DOI:** 10.1038/s41598-017-17195-3

**Published:** 2017-12-04

**Authors:** G. C. Keustermans, D. Kofink, A. Eikendal, W. de Jager, J. Meerding, R. Nuboer, J. Waltenberger, A. O. Kraaijeveld, J. W. Jukema, J. W. Sels, J. Garssen, B. J. Prakken, F. W. Asselbergs, E. Kalkhoven, I. E. Hoefer, G. Pasterkamp, H. S. Schipper

**Affiliations:** 1 Laboratory for Translational Immunology, University Medical Center Utrecht, Utrecht, The Netherlands; 2 Department of Cardiology, Division of Heart and Lungs, University Medical Center Utrecht, Utrecht, The Netherlands; 3 Department of Experimental Cardiology, University Medical Center Utrecht, Utrecht, The Netherlands; 4 Department of Internal medicine, Gastroenterology and Pulmonology, Red Cross Hospital, Beverwijk, The Netherlands; 5 Department of Pediatrics, Meander Medical Center, Amersfoort, The Netherlands; 6 Department of Cardiovascular Medicine, University Hospital Muenster, Muenster, Germany; 7 Department of Cardiology, Leiden University Medical Center, Leiden, The Netherlands; 8 Departments of Cardiology and Intensive Care, Maastricht University Medical Center, Maastricht, The Netherlands; 9 Division of Pharmacology, Utrecht Institute for Pharmaceutical Sciences, Faculty of Science, Utrecht University, Utrecht, The Netherlands; 10 Department of Immunology, Nutricia Research, Utrecht, The Netherlands; 11 Division of Pediatrics, Wilhelmina Children’s Hospital, University Medical Center Utrecht, Utrecht, The Netherlands; 12 Durrer Center for Cardiogenetic Research, ICIN-Netherlands Heart Institute, Utrecht, The Netherlands; 13 Institute of Cardiovascular Science, Faculty of Population Health Sciences, University College London, London, United Kingdom; 14 Molecular Cancer Research and Center for Molecular Medicine, University Medical Center Utrecht, Utrecht, The Netherlands; 15 Department of Pediatric Cardiology, Wilhelmina Children’s Hospital, University Medical Center Utrecht, Utrecht, The Netherlands

## Abstract

Childhood obesity coincides with increased numbers of circulating classical CD14^++^CD16^-^ and intermediate CD14^++^CD16^+^ monocytes. Monocytes are key players in the development and exacerbation of atherosclerosis, which prompts the question as to whether the monocytosis in childhood obesity contributes to atherogenesis over the years. Here, we dissected the monocyte gene expression profile in childhood obesity using an Illumina microarray platform on sorted monocytes of 35 obese children and 16 lean controls. Obese children displayed a distinctive monocyte gene expression profile compared to lean controls. Upon validation with quantitative PCR, we studied the association of the top 5 differentially regulated monocyte genes in childhood obesity with obesity and complexity of coronary atherosclerosis (SYNTAX score) in a cohort of 351 adults at risk for ischemic cardiovascular disease. The downregulation of monocyte IMPDH2 and TMEM134 in childhood obesity was also observed in obese adults. Moreover, downregulation of monocyte TMEM134 was associated with a higher SYNTAX atherosclerosis score in adults. In conclusion, childhood obesity entails monocyte gene expression alterations associated with obesity and enhanced complexity of coronary atherosclerosis in adults.

## Introduction

The childhood obesity epidemic has alarming cardiovascular consequences, and thereby limits the worldwide increase in life expectancy^1,2^. Obesity early in life may contribute to the development of cardiovascular disease in several ways. First, childhood obesity tends to result in adulthood obesity, which is an important risk factor for cardiovascular disease, especially when it concerns visceral adiposity^3,4^. Second, childhood and adulthood obesity share independent risk factors for cardiovascular disease, such as a high blood pressure^5^. Furthermore, obesity-induced insulin resistance and hyperglycemia lead to defective insulin signaling in vascular wall lesional cells, which promotes atherosclerosis at the level of the arterial wall^6^. Finally, obesity is associated with low-grade systemic inflammation, which promotes atherogenesis^7,8^.

At a cellular level, monocytes appear to be a pivotal link between obesity and cardiovascular disease. Obesity is accompanied by leukocytosis, particularly of the myeloid lineage^7,9^. Recent studies indicate that adipose tissue derived inflammatory factors such as IL-1β stimulate bone marrow myeloid progenitors, leading to monocytosis in obesity^10^. Next to increased numbers, monocytes show an activated and inflammatory phenotype in obesity. In humans, monocytes fall into three phenotypical categories: classical CD14^++^CD16^−^, intermediate CD14^++^CD16^+^ and nonclassical CD14^+^CD16^++^ monocytes^11^. Previously, we have shown that childhood obesity is accompanied by increased numbers and an activated phenotype of the classical CD14^++^CD16^−^ monocyte subset^7^. These monocytes are equivalent to GR1^+^Ly6c^high^ monocytes in mice, that differentiate into inflammatory macrophages and foam cells in various atherosclerosis models^12,13^. The increased inflammatory monocyte numbers in childhood obesity may thus contribute to atherogenesis over the years.

The aim of this study was to obtain in-depth understanding of the monocyte gene expression profile in childhood obesity as compared to normal weight controls using micro-array analyses of sorted monocytes. Furthermore, monocyte gene expression profiles were compared with an established cohort of 351 adults at risk for ischemic cardiovascular disease, to study whether monocyte gene expression profiles in childhood obesity overlap with an atherogenic monocyte phenotype in adults. The adult cohort encompassed several clinical parameters, but we focused on the relation between monocyte gene expression and the SYNTAX atherosclerosis score because it is an established angiographic grading system for evaluating the complexity of coronary atherosclerotic lesions, widely used as a readout for atherosclerotic burden^14–18^.

## Results

### Monocytes in childhood obesity show a distinctive gene expression profile

Obese children exhibited typical clinical and biochemical characteristics with a significantly higher Body Mass Index standard deviation for age and sex (BMI-SD) compared to lean controls (3.4 versus 0.4, p < 0.001), a higher systolic blood pressure (BP) (123 mmHg, versus 110 mmHg, p = 0.005), lower Quantitative insulin sensitivity index (QUICKI) (0.3 versus 0.4, p < 0.001) and lower High-density lipoprotein (HDL) cholesterol level (1.2 mmol/L versus 1.5 mmol/L, p = 0.004) (Table 1). Furthermore, the obese subgroup showed a higher total monocyte number (0.6 × 10^9^/ml versus 0.4 × 10^9^/ml, p < 0.001), reflecting a higher classical CD14^++^CD16^−^ monocyte number (52.0 × 10^7^/ml vs. 36.2 × 10^7^/ml, p = 0.001) and a higher intermediate CD14^++^CD16^+^ monocyte number (4.6 × 10^7^/ml versus 3.3 × 10^7^/ml, p < 0.001). Notably, the obese population showed a higher age compared to the lean controls (13.9 versus 10.5 years), and a lower percentage of boys (31% versus 44%). In order to avoid confounding, all subsequent analyses were corrected for age and sex.

**Table 1:** Characteristics of the pediatric study population

	Lean children (n = 16)	Obese children (n = 35)
Age (years)	10.5 (8.4, 12.9)*	13.9 (10.8, 14.9)*
Boys (number, %)	7 (44)	11 (31)
BMI-SD	0.4 (−0.7, 0.9)^§^	3.4 (3.1, 3.7)^§^
QUICKI	0.4 (0.3, 0.4)^§^	0.3 (0.3, 0.3)^§^
Systolic blood pressure (SBP, mmHg)	110 (98, 120)^§^	123 (113, 129)^§^
HDL-cholesterol (mmol/L)	1.5 (1.3, 1.7)^§^	1.2 (1.0, 1.4)^§^
LDL-cholesterol (mmol/L)	2.2 (2.2, 2.4)	2.3 (2.0, 3.0)
Triglycerides (mmol/L)	0.7 (0.5, 0.9)	0.9 (0.7, 1.3)
Total monocyte number (x 10^9^)	0.4 (0.4, 0.5)^§^	0.6 (0.5, 0.7)^§^
CD14^++^CD16^−^ monocyte number (x 10^7^)	36.2 (34.3, 43.2)^§^	52.0 (43.3, 64.7)^§^
CD14^++^CD16^+^ monocyte number (x 10^7^)	1.8 (1.2, 2.3)^§^	4.6 (3.0, 7.0)^§^

Clinical characteristics and laboratory parameters for lean controls versus obese children. Data is shown as median (interquartile range). *p < 0.05, §p < 0.01. BMI-SD: standard deviation of body mass index corrected for age and sex, HDL: high-density lipoprotein, LDL: low-density lipoprotein, QUICKI: quantitative insulin sensitivity index.

**Figure 1:**
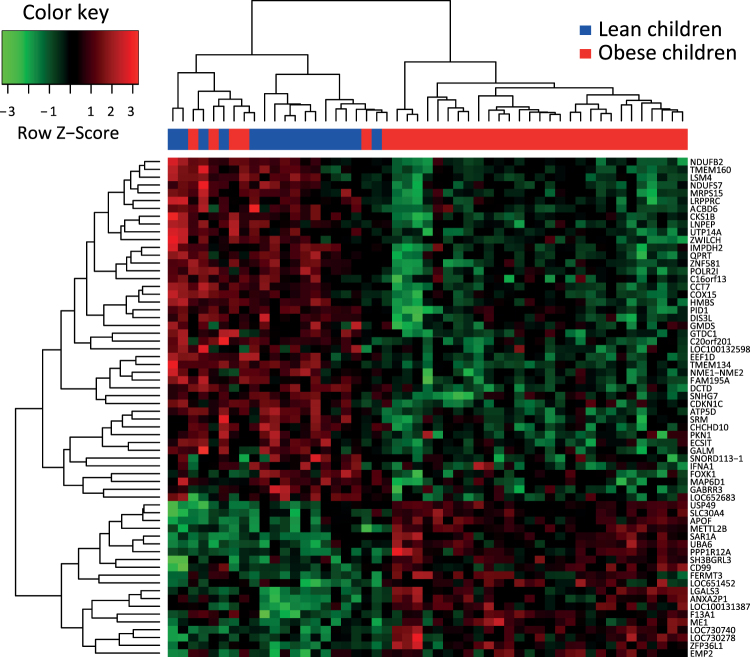
Heat map and cluster analysis of monocyte gene expression. The heat map depicts the gene cluster structure as a hierarchical tree with distinct branches and uses row z-score to depict data that deviates above or below the population mean.

Following microarray multiple testing correction, 67 genes were significantly and differently expressed between the obese and lean participants (Supplemental Tables 1 and 2). An unbiased clustering approach revealed a clear separation in monocyte gene expression profiles between lean and obese individuals (Fig. 1). The microarray data thus highlighted a distinctive monocyte gene expression profile in obese children.

### Quantitative PCR validation

**Figure 2:**
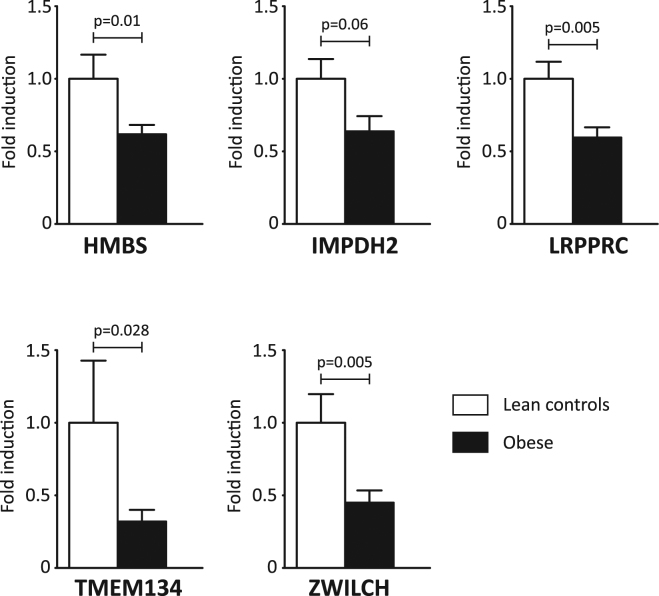
Quantitative PCR confirms downregulation of 5 monocyte genes in childhood obesity. Graphs show fold induction of the gene of interest, normalized for housekeeping gene expression. Error bars represent SEM.

Quantitative PCR (qPCR) was used to confirm the gene expression results, focusing on the top 20 microarray hits. qPCR analyses confirmed the observed downregulation of the monocyte genes Hydroxymethylbilane synthase (HMBS) (p = 0.01), Leucine Rich Pentatricopeptide Repeat Containing (LRPPRC) (p = 0.005), Transmembrane protein 134 (TMEM134) (p = 0.028) and Zwilch Kinetochore Protein (ZWILCH) (p = 0.005) in childhood obesity compared to lean controls (Fig. 2, Supplemental Table 7). Furthermore, Inosine Monophosphate Dehydrogenase 2 (IMPDH2) showed a trend towards downregulation in obese monocytes (p = 0.06). Because of its significant downregulation in monocytes from obese adults, IMPDH2 was included in subsequent analyses as well. Together, these 5 downregulated genes formed the starting point for subsequent studies. First, the association between the 5 downregulated monocyte genes and a selection of clinical variables in childhood was explored (Supplemental Tables 3 and 4). Second, the association with obesity and atherosclerosis was studied in an adult cohort at risk for ischemic cardiovascular disease.

### Pathway analysis

Focusing on the functional relevance of the observed gene expression profile, the functional enrichment of the differentially expressed genes in biological processes was assessed using ToppFun, a GO Term enrichment tool. Sixty-four out of 67 genes could be identified by ToppFun and were mapped to pathways involved in biological processes, using a minimum pathway size of 10 genes. These genes were particularly involved in oxidative phosphorylation and distinct metabolic processes (Supplemental Table 5, Supplemental Fig. 1).

### Monocyte gene expression and adult obesity and cardiovascular risk

To investigate whether adults at risk show a monocyte gene expression profile similar to obese children, the 5 validated monocyte genes were studied in a cohort of 351 adults at risk for ischemic cardiovascular disease. Clinical characteristics of the adult cohort are provided in Supplemental Table 8.

Downregulation of monocyte IMPDH2 (β = −0.496, p = 0.004) and TMEM134 (β = −0.314, p = 0.043) was associated with obesity in adults (BMI >30 kg/m^2^), paralleling our findings in children. These relationships remained significant after adjustment for age and sex (Table 2). Next, we tested whether monocyte gene expression was associated with the established SYNTAX (Synergy between percutaneous coronary intervention with Taxus and Cardiac Surgery) coronary atherosclerosis score. The SYNTAX score uses coronary angiography findings to quantify the complexity of coronary atherosclerosis, based on the number of atherosclerotic lesions, their location and their functional impact^14^. The Syntax score was originally developed to help clinicians select the most appropriate revascularization strategy, but is increasingly being used as a risk stratification tool for adverse ischemic events^14,19^. The SYNTAX score was available for 196 of the 351 adults. Monocyte TMEM134 downregulation was associated with higher SYNTAX scores (β = −0.247, p = 0.041) (Table 3). While adjustment of age and sex did not alter this association (β = −0.251, p = 0.033), addition of BMI to the model attenuated the associated between TMEM134 expression and SYNTAX scores. In summary, monocyte TMEM134 downregulation is observed in childhood obesity, associated with obesity in adults at risk, and correlates with a higher SYNTAX score in adults at risk in an obesity-dependent fashion.

**Table 2:** Monocyte gene expression and obesity in the adult cohort

Gene ID	Transcript	Array ID	*Model* 1	*Model* 2
			β (95% CI)	β (95% CI)
HMBS	ILMN_16358	6060278	−0.213 (−0.505, 0.078)	−0.233 (−0.541, 0.076)
HMBS	ILMN_16358	7320021	−0.194 (−0.496, 0.108)	−0.227 (−0.549, 0.095)
IMPDH2	ILMN_3439	4590026	−0.424 (−0.753, −0.095)*	−0.496 (−0.837, −0.156)^§^
LRPPRC	ILMN_23753	6380064	−0.007 (−0.287, 0.272)	−0.013 (−0.304, 0.277)
TMEM134	ILMN_176754	5690711	−0.061 (−0.318, 0.195)	−0.068 (−0.340, 0.203)
TMEM134	ILMN_183533	670671	−0.311 (−0.610, −0.011)*	−0.314 (−0.620, −0.009)*
ZWILCH	ILMN_166966	7000743	−0.167 (−0.467, 0.133)	−0.168 (−0.485, 0.148)
ZWILCH	ILMN_3475	4850221	−0.247 (−0.556, 0.062)	−0.260 (−0.581, 0.062)

The association of the 5 monocyte genes with obesity in the adult cohort was studied using a logistic regression model. Adult obesity was defined as a BMI >30 kg/m^2^, according to international standards and compared to a normal weight BMI <25 kg/m^2^. Logistic regression coefficients β with 95% confidence intervals (95% CI) for the monocyte genes are shown, both unadjusted (model 1) and adjusted for age and sex (model 2). *p < 0.05, § p < 0.01.

**Table 3:** Monocyte gene expression and SYNTAX score in the adult cohort

Gene ID	Transcript	Array ID	*Model* 1	*Model* 2	*Model* 3
			β (95% CI)	β (95% CI)	β (95% CI)
HMBS	ILMN_16358	6060278	−0.005 (−0.243, 0.232)	0.002 (−0.232, 0.237)	−0.013 (−0.252, 0.227)
HMBS	ILMN_16358	7320021	−0.043 (−0.281, 0.195)	−0.013 (−0.247, 0.221)	−0.030 (−0.268, 0.208)
IMPDH2	ILMN_3439	4590026	−0.133 (−0.370, 0.104)	−0.116 (−0.347, 0.115)	−0.090 (−0.331, 0.150)
LRPPRC	ILMN_23753	6380064	−0.187 (−0.424, 0.049)	−0.188 (−0.419, 0.043)	−0.201 (−0.437, 0.036)
TMEM134	ILMN_176754	5690711	−0.222 (−0.458, 0.014)	−0.211 (−0.443, 0.021)	−0.193 (−0.43, 0.044)
TMEM134	ILMN_183533	670671	−0.247 (−0.483, −0.012)*	−0.251 (−0.481, −0.022)*	−0.227 (−0.464, 0.011)
ZWILCH	ILMN_166966	7000743	−0.102 (−0.339, 0.136)	−0.171 (−0.404, 0.062)	−0.168 (−0.408, 0.071)
ZWILCH	ILMN_3475	4850221	0.030 (−0.207, 0.268)	0.010 (−0.223, 0.242)	0.048 (−0.197, 0.293)

The association of the 5 validated genes with severity of coronary artery atherosclerosis (SYNTAX score, square-root transformed) was studied using linear regression. Linear regression coefficients β with 95% confidence intervals (95% CI) for the monocyte genes are shown. Model 1: unadjusted, model 2: adjusted for age and sex, model 3: adjusted for age, sex and BMI. *p < 0.05.

## Discussion

Monocytes are key players in the development and exacerbation of atherosclerosis, both via their role as macrophage foam cell precursors and their role in systemic inflammation^20^. In human studies, increased numbers of classical CD14^++^CD16^−^ and intermediate CD14^++^CD16^+^ monocytes predict cardiovascular events independent of age, sex and classical cardiovascular risk factors^21,22^. Interestingly, childhood obesity also coincides with increased circulating numbers of classical and intermediate monocytes^7^, which prompts the question as to whether the monocytosis in childhood obesity contributes to atherogenesis over the years. The atherogenic role of monocytes in childhood obesity is difficult to study in human models, since longitudinal data are lacking. To the best of our knowledge, this is the first monocyte gene expression study in childhood obesity, and the first endeavor to crosscheck gene expression profiles in adults at risk for ischemic cardiovascular disease. Our study showed a distinctive monocyte gene expression profile in childhood obesity, and downregulation of monocyte IMPDH2 and TMEM134 was also associated with obesity in the adult cohort at risk. Finally, downregulated TMEM134 coincided with a higher SYNTAX score in adults at risk, reflecting an enhanced atherosclerotic burden^14,19^.

Our results stress the relevance of the monocytosis in childhood obesity and raise several interesting questions. First, pathway analysis of differentially regulated monocyte genes in childhood obesity revealed an overrepresentation of oxidative phosphorylation, oxidative stress, and intracellular metabolism pathways, which apparently reflects reprogramming to aerobic glycolysis. While resting immune cells primarily need ATP to meet cellular demands, and use glucose-pyruvate conversion (glycolysis) and oxidative phosphorylation to fulfill these needs, many immune cells in inflammatory microenvironments undergo metabolic reprogramming to aerobic glycolysis in order to engage in cellular growth and proliferation^23^. Interestingly, an upregulation of aerobic glycolysis also coincides with the development of ‘trained immunity’^24^. Upon repetitive stimulation with microbial moieties and/or metabolites, monocytes undergo epigenetic reprogramming towards aerobic glycolysis, which enhances the response of the trained monocytes in case of restimulation^23,25^. Recent literature suggests that the development of trained immunity contributes to the development of systemic inflammation and atherosclerosis, and represents an intriguing target for therapeutic intervention^25^.

Second, the role of TMEM134 in monocytes gains traction. In human monocyte studies, the expression of TMEM134 was decreased in classical CD14^++^CD16^−^ and intermediate CD14^++^CD16^+^ monocytes, in contrast to nonclassical CD14^+^CD16^++^ monocytes^26^. Hence TMEM134 downregulation in childhood obesity and adults with cardiovascular risk may reflect obesity-induced CD14^++^CD16^-^ and CD14^++^CD16^+^ monocytosis. Whether the highly conserved 21.5 kDa transmembrane protein TMEM134 plays an active role in monocyte differentiation remains to be elucidated. Notably, the existing studies indicate that TMEM134 affects the prototypical inflammatory nuclear factor-κB (NF-κB) signaling pathway. TMEM 134 was identified as a binding protein of latent membrane protein 1 (LMP1) and Hepatitis E Virus Open Reading Frame 2 (ORF2), and affected downstream NF-κB signalling via these binding partners^27,28^. Importantly, modulation of downstream NF-κB signalling is considered one of the hallmarks of innate immune programming in chronic inflammation^29^. Therefore, it is tempting to speculate that the observed downregulation of TMEM134 in childhood obesity monocytes is connected to the development of trained immunity, as discussed previously.

Finally, limitations of the current study have to be taken into account. Since our pediatric study population was relatively small, the associations reported in our study are of subtle strength. Second, environmental factors such as freeze-thawing of the monocytes may have influenced gene expression profiles. Though pediatric and adult samples were treated similarly, minor processing differences could impact gene expression profiles. Third, we chose to focus on the 5 qPCR-validated monocyte genes. Thereby, we may have disregarded important monocyte genes that were not included in the qPCR validation. Finally, CD14-positive magnetic bead sorting skewed the analyzed monocyte compartments towards classical CD14^++^CD16^−^ and intermediate CD14^++^CD16^+^ monocytes (Supplemental Fig. 2), and partly disregarded the nonclassical CD14^+^CD16^++^ monocyte subset, which is considered less important for atherosclerosis development^21,22^.

In conclusion, childhood obesity entails monocyte gene expression alterations associated with obesity and enhanced complexity of coronary atherosclerosis in adults. Especially the role of TMEM134 in monocytes gains traction, as downregulation of monocyte TMEM134 was associated with obesity in children and adults, and coincided with a higher SYNTAX atherosclerosis score in adults at risk for ischemic cardiovascular disease.

## Methods

### Pediatric cohort

Peripheral blood mononuclear cells (PBMC) were studied of 51 children aged 6–16 years (35 obese, 16 lean controls). The cells were derived from a previously published cross-sectional study at the Pediatric Outpatient Department of the Meander Medical Center in Amersfoort, the Netherlands, consisting of 60 obese children and 30 age- and sex-matched lean controls^7^. Because PBMC were available for 35 obese children and 16 lean controls, these children were included in the current study. Importantly, the availability of stored PBMC depended on the amount of blood a patient donated upon inclusion, which varied randomly. Therefore, we believe patients in the current study are a random selection of the previous study.

BMI-SD was calculated using the outcomes of the Fifth Dutch Growth Study (2008–2010). Childhood obesity was defined as BMI-SD >2.5, which can be extrapolated toward the international definition of obesity as BMI >30 kg/m2 for adults^30,31^. Blood pressure was measured using an automated oscillometric method (Dinamap; GE Healthcare, Amersham, UK). Lipid profiles where obtained using standardized laboratory procedures. Written informed consent was obtained from all children and their parents. The study was approved by the Institutional Medical Ethical Review Board of the University Medical Center Utrecht, The Netherlands. All experiments with human biological materials were performed in accordance with the relevant guidelines and regulations.

### Adult cohort

CTMM Circulating Cells is a multi-center cohort of four Dutch medical centers that enrolled patients with stable or unstable angina pectoris undergoing coronary angiography, with the aim of identifying cellular biomarkers for the prediction of adverse cardiovascular events. Patients were recruited between March 2009 and September 2011. Details of the study design have been described elsewhere^32^. All participants provided written informed consent. The study was approved by the Institutional Medical Ethical Review Board of the University Medical Center Utrecht, The Netherlands. Data from 351 patients were included in the final analysis after removal of samples with outlying median intensity (Supplemental Table 8). Gene expression profiles were quantile-normalized followed by log2 transformation. The complexity of coronary atherosclerosis was assessed with coronary angiography using the SYNTAX score system. Two independent observers quantified SYNTAX scores, using SYNTAX score calculator version 2.11. The SYNTAX score is a tool for evaluating the complexity of coronary artery disease, taking into account the number of atherosclerotic lesions, their location and their functional impact^14^. SYNTAX scores were available from 196 of the 351 patients.

### Monocytes

In both cohorts, peripheral blood mononuclear cells (PBMC) were isolated using Ficoll-Paque density gradient centrifugation. In the pediatric cohort, flow cytometric phenotyping was performed in earlier studies^7^. Subsequent to isolation, samples were stored in freeze medium (FCS with 10% DMSO, Sigma-Aldrich) until further use. In order to isolate monocytes, stored samples where thawed and washed in medium comprising of RPMI1640 supplemented with l-glutamate and 25 mM HEPES (Gibco), containing 2% FCS and penicillin/streptomycin (100 U/mL) (Invitrogen). Cells where spun down for 10 min, 1600 rpm at room temperature. PBMCs where then resuspended in MACS buffer - 2%FBS (Biowest), 2%EDTA (VWR chemicals) in PBS (Gibco) - and counted using the trypan blue exclusion method (Gibco). Anti-human CD14 magnetic particles where subsequently used to isolate monocytes using the company protocol (BD IMag). The CD14 positive cells were then re-suspended in 500ul of TRIZOL (Life Technologies) and stored at −80 °C.

### Microarray and data processing

RNA was isolated from the trizol-lysed samples by AROS Applied Biotechnology. Samples of the 35 obese children and 16 healthy control children and the 351 adults underwent the same isolation procedures, and were similarly processed. In short, samples were labeled using the Illumina TotalPrep RNA Amplification Kit and 100 ng of total RNA. The IVT product was QC-checked on gel and quantitated using the NanoDrop (Thermo Scientific). 750 ng of cDNA was used for the standard Illumina protocol before samples were hybridized on the arrays (Illumina humanHT-12 v3). Arrays were scanned using a Bead Array Reader (Illumina). After inspection of the sample median intensities, samples with a median intensity of <50 were removed. Subsequently, the expression data was quantile-normalised and log2 transformed using the lumi R package^33^.

### qPCR Validation

To validate the 67 differentially expressed genes, qPCR primers were designed for the top 20 hits. Of these primer pairs 17 functioned optimally and were deemed applicable for the validation process (Supplementary Table 6). High quality RNA of 27 obese children and 11 healthy controls was available for the qPCR validation studies. qPCR analysis was performed using SYBR Select Master Mix reagents (Thermo Fischer Scientific) and run using the QuantStudio Flex system (Thermo Fischer Scientific). Data was normalized for housekeeping gene expression of GUSB, 36B4 and B2M, in accordance with international standards^34^.

### Statistics

First, demographic characteristics of the study population were presented as numbers and percentages for categorical variables and as means with standard deviation (SD) or medians with interquartile ranges (Q1, Q3) for normal and non-normally distributed continuous variables, respectively. Subsequently, monocyte gene expression profiles of lean and obese children were compared and significant differences between both groups were assessed using Mann Whitney U tests for continuous variables and X^2^ test for binary variables.

Second, monocyte gene expression profiles were compared using the Limma package in R. In short, the Limma package uses empirical Baysian methods for the analysis of gene expression microarray data and is specifically designed for analyzing smaller datasets^33^. For this analysis, the genes functioned as outcome variables (dependent variables) and obesity status as determinant (independent variable). Age and sex were included in the model as covariates. In addition, to adjust for multiple testing, Benjamini Hochberg (BH) correction was applied. To illustrate the results of the microarray analysis, a heat map was generated using the heatmap.2 function in R. Hierarchical clustering was performed using complete linkage.

Third, linear regression was used to study the relation between obesity status and the gene expression (dependent variable) For this analysis, two models were constructed; a crude model (Model 1) and a model in which age and sex were included as covariates (Model 2) (Supplemental Table 2).

Fourth, the relation between the 5 qPCR validated genes and clinical variables was studied in the whole pediatric cohort (*n* = 51) as well as the obese subgroup (*n = *35) and the adult cohort (*n = *351) using linear regression analysis (Supplemental Tables 3 and 4). For the analysis performed in the whole pediatric cohort age, sex and BMI-SD were included as covariates whereas for the analysis in the obese subgroup age and sex were included as covariates. P_2-sided_ <0.05 was considered statistically significant.

### Data availability

The pediatric datasets generated and analyzed during the current study are available from the corresponding author on reasonable request. Restrictions apply to data of the adult CTMM Circulating Cells dataset, which were used under license for the current study, and are not publicly available. Data are available from the authors upon reasonable request and after permission of the CTMM Circulating Cells consortium.

## Electronic supplementary material

Supplemental data
