# Supplementary material for: Monocyte gene expression in childhood obesity is associated with obesity and complexity of atherosclerosis in adults
Source: Sci Rep. 2017 Dec 4;7:16826. doi: 10.1038/s41598-017-17195-3 (PMC5714995; doi:10.1038/s41598-017-17195-3)
Supplement: Supplementary file 1 — Supplemental data [file 41598_2017_17195_MOESM1_ESM.pdf]

Supplemental data

Monocyte gene expression in childhood obesity is associated with obesity and complexity of atherosclerosis in adults

Keustermans GC, Kofink D, Eikendal A, de Jager W, Meerding J, Nuboer R, Waltenberger J, Kraaijeveld AO, Jukema JW, Sels JW, Garssen J, Prakken BJ, Asselbergs FW, Kalkhoven E, Hoefer IE, Pasterkamp G, Schipper HS

Supplemental table 1 Significantly different genes between obese and lean children in total study population (n = 51)

	Log Fold change	Crude p-value	FDR-adjusted p-value	Beta
**ACBD6**	-0.16	<0.0001	0.03	3.35
**ANXA2P1**	0.25	<0.0001	0.04	1.75
**APOF**	0.23	<0.0001	0.03	2.07
**ATP5D**	-0.16	<0.0001	0.03	2.31
**C16orf13**	-0.17	<0.0001	0.05	1.33
**C20orf201**	-0.15	<0.0001	0.04	1.98
**CCT7**	-0.29	<0.0001	0.03	2.47
**CD99**	0.29	<0.0001	0.04	1.85
**CDKN1C**	-0.41	<0.0001	0.05	1.32
**CHCHD10**	-0.17	<0.0001	0.04	1.86
**CKS1B**	-0.30	<0.0001	0.03	2.46
**COX15**	-0.32	<0.0001	0.04	1.57
**DCTD**	-0.14	<0.0001	0.03	2.88
**DIS3L**	-0.32	<0.0001	0.04	1.92
**ECSIT**	-0.21	<0.0001	0.03	3.69
**EEF1D**	-0.17	<0.0001	0.03	3.04
**EMP2**	0.14	<0.0001	0.03	2.36
**F13A1**	0.62	<0.0001	0.03	3.70
**FAM195A**	-0.35	<0.0001	0.03	2.42
**FERMT3**	0.11	<0.0001	0.03	3.29
**FOXK1**	-0.19	<0.0001	0.04	1.89
**GABRR3**	-0.09	<0.0001	0.03	2.57
**GALM**	-0.17	<0.0001	0.04	1.47
**GMDS**	-0.20	<0.0001	0.04	1.57
**GTDC1**	-0.10	<0.0001	0.04	1.73
**HMBS**	-0.27	<0.0001	0.03	2.76
**IFNA1**	-0.09	<0.0001	0.03	2.07
**IMPDH2**	-0.28	<0.0001	0.02	4.98
**LGALS3**	0.31	<0.0001	0.03	2.80
**LNPEP**	-0.12	0.0001	0.05	1.22
**LOC100130604**	0.36	<0.0001	0.03	2.18
**LOC100131387**	0.16	<0.0001	0.03	2.19
**LOC100132598**	-0.08	0.0001	0.05	1.19
**LOC651452**	0.07	<0.0001	0.03	2.38
**LOC652683**	-0.10	<0.0001	0.03	2.10
**LOC653778**	0.40	<0.0001	0.04	1.84
**LOC654121**	0.20	<0.0001	0.03	2.65
**LOC730278**	0.31	<0.0001	0.03	2.51
**LOC730740**	0.16	<0.0001	0.03	3.24
**LRPPRC**	-0.21	<0.0001	0.03	3.15
**LSM4**	-0.23	0.0001	0.05	1.20
**MAP6D1**	-0.33	<0.0001	0.03	2.78

**ME1**	0.27	<0.0001	0.02	4.37
**METTL2B**	0.14	<0.0001	0.03	2.86
**MRPS15**	-0.16	<0.0001	0.05	1.30
**NDUFB2**	-0.22	<0.0001	0.04	1.63
**NDUFS7**	-0.23	<0.0001	0.05	1.28
**NME1-NME2**	-0.23	<0.0001	0.03	3.42
**PID1**	-0.44	<0.0001	0.03	2.07
**PKN1**	-0.15	<0.0001	0.03	2.07
**POLR2I**	-0.20	0.0001	0.05	1.22
**PPP1R12A**	0.25	<0.0001	0.04	1.48
**QPRT**	-0.32	<0.0001	0.00	8.56
**SAR1A**	0.32	<0.0001	0.04	1.75
**SH3BGRL3**	0.26	<0.0001	0.03	3.24
**SLC30A4**	0.19	<0.0001	0.03	2.48
**SNHG7**	-0.39	<0.0001	0.02	4.61
**SNORD113-1**	-0.08	<0.0001	0.04	1.73
**SRM**	-0.17	<0.0001	0.04	1.42
**TMEM134**	-0.19	<0.0001	0.02	4.54
**TMEM160**	-0.28	<0.0001	0.04	1.77
**UBA6**	0.36	<0.0001	0.04	1.53
**USP49**	0.55	<0.0001	0.04	1.56
**UTP14A**	-0.16	<0.0001	0.05	1.37
**ZFP36L1**	0.40	<0.0001	0.04	1.96
**ZNF581**	-0.17	<0.0001	0.04	1.51
**ZWILCH**	-0.12	<0.0001	0.03	2.62

Supplemental table 2 Association between genes and obesity status in total study population (n = 51)

	Model 1*† β (95% CI)	p value	Model 2*† β (95% CI)	p value
**ACBD6**	-0.16 (-0.22, -0.10)	<0.0001	-0.16 (-0.22, -0.09)	<0.0001
**ANXA2P1**	0.26 (0.16, 0.37)	<0.0001	0.25 (0.13, 0.36)	<0.0001
**APOF**	0.22 (0.12, 0.32)	<0.0001	0.23 (0.12, 0.33)	<0.0001
**ATP5D**	-0.17 (-0.23, -0.10)	<0.0001	-0.16 (-0.23, -0.09)	<0.0001
**C16orf13**	-0.15 (-0.23, -0.07)	0.0004	-0.17 (-0.26, -0.09)	<0.0001
**C20orf201**	-0.16 (-0.22, -0.09)	<0.0001	-0.15 (-0.22, -0.08)	0.0001
**CCT7**	-0.28 (-0.40, -0.16)	<0.0001	-0.29 (-0.42, -0.16)	<0.0001
**CD99**	0.26 (0.13, 0.39)	0.0002	0.29 (0.16, 0.43)	<0.0001
**CDKN1C**	-0.40 (-0.59, -0.22)	<0.0001	-0.41 (-0.61, -0.21)	0.0001
**CHCHD10**	-0.19 (-0.26, -0.11)	<0.0001	-0.17 (-0.25, -0.09)	<0.0001
**CKS1B**	-0.28 (-0.41, -0.16)	<0.0001	-0.30 (-0.43, -0.16)	<0.0001
**COX15**	-0.29 (-0.43, -0.15)	<0.0001	-0.32 (-0.47, -0.17)	0.0001
**DCTD**	-0.16 (-0.22, -0.11)	<0.0001	-0.14 (-0.20, -0.08)	<0.0001
**DIS3L**	-0.31 (-0.45, -0.18)	<0.0001	-0.32 (-0.46, -0.17)	<0.0001
**ECSIT**	-0.21 (-0.29, -0.13)	<0.0001	-0.21 (-0.30, -0.13)	<0.0001
**EEF1D**	-0.15 (-0.22, -0.08)	<0.0001	-0.17 (-0.24, -0.10)	<0.0001
**EMP2**	0.12 (0.06, 0.18)	0.0002	0.14 (0.08, 0.20)	<0.0001
**F13A1**	0.64 (0.41, 0.88)	<0.0001	0.62 (-0.37, 0.88)	<0.0001
**FAM195A**	-0.34 (-0.48, -0.19)	<0.0001	-0.35 (-0.51, -0.20)	<0.0001
**FERMT3**	0.11 (0.07, 0.15)	<0.0001	0.11 (0.07, 0.15)	<0.0001
**FOXK1**	-0.19 (-0.27, -0.11)	<0.0001	-0.19 (-0.28, -0.10)	<0.0001
**GABRR3**	-0.08 (-0.12, -0.05)	<0.0001	-0.09 (-0.13, -0.05)	<0.0001
**GALM**	-0.18 (-0.26, -0.11)	<0.0001	-0.17 (-0.26, -0.09)	0.0001
**GMDS**	-0.18 (-0.27, -0.09)	0.0001	-0.20 (-0.29, -0.10)	<0.0001
**GTDC1**	-0.10 (-0.14, -0.06)	<0.0001	-0.10 (-0.15, -0.06)	<0.0001
**HMBS**	-0.25 (-0.36, -0.15)	<0.0001	-0.27 (-0.38, -0.15)	<0.0001
**IFNA1**	-0.08 (-0.11, -0.04)	0.0003	-0.09 (-0.13, -0.05)	<0.0001
**IMPDH2**	-0.29 (-0.39, -0.19)	<0.0001	-0.28 (-0.39, -0.18)	<0.0001
**LGALS3**	0.35 (0.22, 0.48)	<0.0001	0.31 (0.18, 0.44)	<0.0001
**LNPEP**	-0.13 (-0.18, -0.07)	<0.0001	-0.12 (-0.18, -0.06)	0.0001
**LOC100130604**	0.38 (0.23, 0.53)	<0.0001	0.36 (0.20, 0.53)	<0.0001
**LOC100131387**	0.17 (0.11, 0.24)	<0.0001	0.16 (0.09, 0.23)	<0.0001
**LOC100132598**	-0.08 (-0.11, -0.04)	<0.0001	-0.08 (-0.12, -0.04)	<0.0001
**LOC651452**	0.06 (0.04, 0.09)	<0.0001	0.07 (0.04, 0.10)	<0.0001
**LOC652683**	-0.09 (-0.13, -0.05)	<0.0001	-0.10 (-0.14, -0.05)	<0.0001
**LOC653778**	0.39 (0.21, 0.56)	<0.0001	0.40 (0.21, 0.59)	<0.0001
**LOC654121**	0.20 (0.13, 0.28)	<0.0001	0.20 (-0.11, 0.28)	<0.0001
**LOC730278**	0.32 (0.19, 0.45)	<0.0001	0.31 (0.18, 0.45)	<0.0001
**LOC730740**	0.17 (0.11, 0.23)	<0.0001	0.16 (0.09, 0.23)	<0.0001
**LRPPRC**	-0.21 (-0.30, -0.13)	<0.0001	-0.21 (-0.30, -0.12)	<0.0001
**LSM4**	-0.22 (-0.33, -0.12)	<0.0001	-0.23 (-0.34, -0.12)	0.0001
**MAP6D1**	-0.36 (-0.49, -0.23)	<0.0001	-0.33 (-0.48, -0.19)	<0.0001

**ME1**	0.27 (0.17, 0.38)	<0.0001	0.27 (0.17, 0.38)	<0.0001
**METTL2B**	0.13 (0.08, 0.19)	<0.0001	0.14 (0.08, 0.21)	<0.0001
**MRPS15**	-0.17 (-0.24, -0.10)	<0.0001	-0.16 (-0.24, -0.08)	0.0001
**NDUFB2**	-0.22 (-0.32, -0.13)	<0.0001	-0.22 (-0.33, -0.12)	<0.0001
**NDUFS7**	-0.24 (-0.34, -0.14)	<0.0001	-0.23 (-0.34, -0.12)	0.0001
**NME1-NME2**	-0.23 (-0.31, -0.14)	<0.0001	-0.23 (-0.32, -0.13)	<0.0001
**PID1**	-0.43 (-0.62, -0.25)	<0.0001	-0.44 (-0.65, -0.24)	<0.0001
**PKN1**	-0.14 (-0.20, -0.08)	<0.0001	-0.15 (-0.22, -0.08)	<0.0001
**POLR2I**	-0.21 (-0.30, -0.12)	<0.0001	-0.20 (-0.30, -0.11)	0.0001
**PPP1R12A**	0.27 (0.16, 0.38)	<0.0001	0.25 (0.13, 0.37)	0.0001
**QPRT**	-0.31 (-0.40, -0.22)	<0.0001	-0.32 (-0.42, -0.22)	<0.0001
**SAR1A**	0.33 (0.19, 0.46)	<0.0001	0.32 (0.17, 0.47)	<0.0001
**SH3BGRL3**	0.25 (0.14, 0.36)	<0.0001	0.26 (0.15, 0.38)	<0.0001
**SLC30A4**	0.16 (0.09, 0.24)	<0.0001	0.19 (0.11, 0.27)	<0.0001
**SNHG7**	-0.37 (-0.51, -0.24)	<0.0001	-0.39 (-0.54, -0.24)	<0.0001
**SNORD113-1**	-0.07 (-0.10, -0.03)	0.0003	-0.08 (-0.12, -0.05)	<0.0001
**SRM**	-0.18 (-0.25, -0.10)	<0.0001	-0.17 (-0.25, -0.09)	<0.0001
**TMEM134**	-0.19 (-0.26, -0.13)	<0.0001	-0.19 (-0.26, -0.12)	<0.0001
**TMEM160**	-0.28 (-0.40, -0.16)	<0.0001	-0.28 (-0.42, -0.15)	<0.0001
**UBA6**	0.33 (0.17, 0.49)	0.0002	0.36 (0.19, 0.54)	0.0001
**USP49**	0.55 (0.31, 0.79)	<0.0001	0.55 (0.29, 0.81)	0.0001
**UTP14A**	-0.14 (-0.21, -0.07)	0.0002	-0.16 (-0.23, -0.08)	0.0001
**ZFP36L1**	0.44 (0.27, 0.62)	<0.0001	0.40 (0.22, 0.59)	<0.0001
**ZNF581**	-0.18 (-0.25, -0.10)	<0.0001	-0.17 (-0.25, -0.09)	<0.0001
**ZWILCH**	-0.11 (-0.16, -0.06)	<0.0001	-0.12 (-0.17, -0.07)	<0.0001

* Model 1: crude, Model 2: adjusted for age and sex (reference category: lean)

† Values are linear regression coefficients (β) with 95% confidence intervals (CI)

Supplemental table 3 Monocyte gene expression and cardiometabolic parameters in the total pediatric population

		Genes HMBS	IMPDH2	LRPPRC	TMEM134	ZWILCH
Cardiovascular risk factors						
Systolic BP	β	-2.52	11.46	-0.66	-3.13	-26.43
	95% CI	-18.87, 13.84	-6.47, 29.38	-21.39, 20.07	-29.94, 23.67	-63.45, 10.59
QUICKI	β	-0.01	0.03	-0.06	0.06	-0.02
	95% CI	-0.06, 0.05	-0.02, 0.09	-0.13, 0.001	-0.02, 0.14	-0.14, 0.10
Monocytes						
Total monocyte nr	β	-0.06	-0.25	-0.15	-0.18	-0.51
	95% CI	-0.33, 0.20	-0.54, 0.04	-0.49, 0.18	-0.61, 0.24	-1.11, 0.09
$CD14^{++}CD16^{-}$ nr	β	-5.08	-25.98	-12.28	-25.81	-54.99
	95% CI	-32.18, 22.01	-55.11, 3.15	-46.14, 21.58	-68.19, 16.56	-114.69, 4.71
$CD14^{++}CD16^{+}$ nr	β	-0.26	-2.16	-5.55	2.48	-7.97
	95% CI	-6.86, 6.34	-9.47, 5.15	-13.66, 2.56	-7.98, 12.93	-22.85, 6.91
$CD14^{++}CD16^{-}$	β	-2442	-3071	-10184 *	-5021.0	-19312 *
CD11b MFI	95% CI	-10380, 5495	-12069, 5927	-18305, -2062	-18839, 8797	-36097, -2527
$CD14^{++}CD16^{+}$	β	362	-1705	-6765	2012	-8736
CD11b MFI	95% CI	-7767, 8491	-1094, 7507	-15456, 1925	-12154, 16178	-26844, 9373
Adipokines						
Adiponectin	β	13.56	10.41	10.33	20.66	36.01 *
	95% CI	-1.79, 28.90	-7.67, 28.49	-9.17, 29.83	-3.96, 45.29	1.43, 70.60
Chemerin	β	-0.84 *	-0.25	-0.27	-1.24 *	-0.14
	95% CI	-1.60, -0.09	-1.13, 0.63	-1.26, 0.76	-2.45, -0.03	-1.96, 1.68
Leptin	β	-7.05	106.93	27.60	116.45	311.12
	95% CI	-204.94, 190.85	-110.29, 324.16	-220.06, 275.26	-195.93, 428.84	-130.88, 753.11
TNF-R2	β	0.11	1.01 *	0.48	1.02	1.86
	95% CI	-0.77, 0.99	0.08, 1.94	-0.62, 1.57	-0.34, 2.39	-0.07, 3.79

The association of the 5 validated genes with a selection of cardiometabolic parameters was studied using a linear regression model. Linear regression coefficients (β) with 95% confidence intervals (95% CI) for the monocyte genes are shown, adjusted for age, sex and BMI-SD. *p<0.05, § p<0.01. MFI: median fluorescence intensity, nr: number, QUICKI: quantitative insulin sensitivity index, Systolic BP: systolic blood pressure.

Supplemental table 4 Monocyte gene expression and cardiometabolic parameters in the obese pediatric subgroup

		Genes HMBS	IMPDH2	LRPPRC	TMEM134	ZWILCH
Cardiovascular risk factors						
Systolic BP	β	-1.41	20.72 *	11.22	-5.82	-25.87
	95% CI	-19.59, 16.78	1.25, 40.19	-15.87, 38.30	-38.63, 26.99	-74.00, 22.26
QUICKI	β	-0.03	-0.03	-0.08 *	-0.01	-0.14 *
	95% CI	-0.09, 0.02	-0.09, 0.04	-0.16, -0.001	-0.12, 0.09	-0.28, -0.001
Monocytes						
Total monocyte nr	β	0.02	-0.17	-0.05	0.02	-0.35
	95% CI	-0.37, 0.41	-0.61, 0.28	-0.64, 0.54	-0.69, 0.72	-1.39, 0.70
$CD14^{++}CD16^{-}$ nr	β	4.15	1.09	-3.80	12.66	-6.53
	95% CI	-5.81, 14.11	-10.44, 12.62	-18.78, 11.18	-4.75, 30.07	-33.05, 19.98
$CD14^{++}CD16^{+}$ nr	β	6.98	-15.09	1.94	-4.53	-36.60
	95% CI	-33.07, 47.02	-60.64, 30.46	-57.94, 61.82	-76.39, 67.34	-141.66, 68.47
$CD14^{++}CD16^{-}$	β	3389	-8455	-15385 *	10387	-24070
CD11b MFI	95% CI	-9927, 16705	-12017, 14902	-29859, -912	-18329, 39102	-62508, 14369
$CD14^{++}CD16^{+}$	β	2323	-839	-19214 §	17823	-23782
CD11b MFI	95% CI	-11681, 16326	-14954, 13275	-33658, -4771	-11611, 47257	-64228, 16665
Adipokines						
Adiponectin	β	16.47	7.72	-0.55	16.56	23.05
	95% CI	-0.54, 33.48	-13.85, 29.29	-27.07, 26.01	-16.04, 49.17	-24.31, 70.40
Chemerin	β	-1.09 *	-0.17	-0.28	-2.68 §	-1.17
	95% CI	-2.14, -0.03	-1.46, 1.12	-1.95, 1.40	-4.45, -0.92	-4.13, 1.78
Leptin	β	580	238	144	377	759
	95% CI	-265, 380	-120, 596	-333, 621	-186, 939	-46, 1563
TNF-R2	β	0.16	1.28 *	0.58	1.17	1.19
	95% CI	-0.99, 1.30	0.07, 2.49	-1.10, 2.26	-0.83, 3.17	-1.80, 4.17

The association of the 5 validated genes with a selection of cardiometabolic parameters was studied using a linear regression model. Linear regression coefficients (β) with 95% confidence intervals (95% CI) for the monocyte genes are shown, adjusted for age and sex. *p<0.05, § p<0.01. MFI: median fluorescence intensity, nr: number, QUICKI: quantitative insulin sensitivity index, Systolic BP: systolic blood pressure.

Supplemental table 5 Pathway analysis

Name	Gene ID	Hits in query list	Number of genes in pathway	Raw p-value	P-value (FDR-adjusted)	Hits in Query List
oxidative phosphorylation	GO:0006119	5	108	1,58E-05	4,24E-03	ATP5D,CHCHD10,NDUFS7,COX15,NDUFB2
purine ribonucleoside monophosphate metabolic process	GO:0009167	7	279	1,63E-05	4,24E-03	ATP5D,CHCHD10,NDUFS7,IMPDH,COX15,NDUFB2,PID1
purine nucleoside monophosphate metabolic process	GO:0009126	7	280	1,67E-05	4,24E-03	ATP5D,CHCHD10,NDUFS7,IMPDH,COX15,NDUFB2,PID1
ribonucleoside monophosphate metabolic process	GO:0009161	7	291	2,14E-05	4,24E-03	ATP5D,CHCHD10,NDUFS7,IMPDH,COX15,NDUFB2,PID1
nucleoside metabolic process	GO:0009116	8	414	2,56E-05	4,24E-03	ATP5D,CHCHD10,NDUFS7,IMPDH,COX15,DCTD,NDUFB2,PID1
nucleoside monophosphate metabolic process	GO:0009123	7	304	2,83E-05	4,24E-03	ATP5D,CHCHD10,NDUFS7,IMPDH,COX15,NDUFB2,PID1
nucleotide metabolic process	GO:0009117	10	694	2,88E-05	4,24E-03	ATP5D,CHCHD10,NDUFS7,IMPDH,QPRT,COX15,DCTD,NDUFB2,ME1,PID1
nucleoside phosphate metabolic process	GO:0006753	10	704	3,26E-05	4,24E-03	ATP5D,CHCHD10,NDUFS7,IMPDH,QPRT,COX15,DCTD,NDUFB2,ME1,PID1
glycosyl compound metabolic process	GO:1901657	8	436	3,70E-05	4,24E-03	ATP5D,CHCHD10,NDUFS7,IMPDH,COX15,DCTD,NDUFB2,PID1
nucleoside biosynthetic process	GO:0009163	5	131	4,02E-05	4,24E-03	ATP5D,CHCHD10,IMPDH2,DCTD,PID1
nucleotide biosynthetic process	GO:0009165	7	322	4,08E-05	4,24E-03	ATP5D,CHCHD10,IMPDH2,QPRT,DCTD,ME1,PID1
glycosyl compound biosynthetic process	GO:1901659	5	133	4,32E-05	4,24E-03	ATP5D,CHCHD10,IMPDH2,DCTD,PID1
nucleoside phosphate biosynthetic process	GO:1901293	7	325	4,33E-05	4,24E-03	ATP5D,CHCHD10,IMPDH2,QPRT,DCTD,ME1,PID1
organonitrogen compound biosynthetic process	GO:1901566	14	1445	5,17E-05	4,69E-03	ATP5D,LRPPRC,EEF1D,CHCHD10,IMPDH2,ZFP36L1,QPRT,MRPS15, SRM,HMBS,COX15,DCTD,ME1,PID1
purine nucleoside monophosphate biosynthetic process	GO:0009127	4	74	6,50E-05	4,96E-03	ATP5D,CHCHD10,IMPDH2,PID1
purine ribonucleoside monophosphate biosynthetic process	GO:0009168	4	74	6,50E-05	4,96E-03	ATP5D,CHCHD10,IMPDH2,PID1
nucleobase-containing small molecule metabolic process	GO:0055086	10	766	6,63E-05	4,96E-03	ATP5D,CHCHD10,NDUFS7,IMPDH2,QPRT,COX15,DCTD,NDUFB2,ME1,PID1
ATP metabolic process	GO:0046034	6	242	7,35E-05	5,19E-03	ATP5D,CHCHD10,NDUFS7,COX15,NDUFB2,PID1
purine ribonucleoside metabolic process	GO:0046128	7	362	8,53E-05	5,71E-03	ATP5D,CHCHD10,NDUFS7,IMPDH2,COX15,NDUFB2,PID1
purine nucleoside metabolic process	GO:0042278	7	365	8,98E-05	5,71E-03	ATP5D,CHCHD10,NDUFS7,IMPDH2,COX15,NDUFB2,PID1
purine ribonucleoside triphosphate metabolic process	GO:0009205	6	263	1,16E-04	6,76E-03	ATP5D,CHCHD10,NDUFS7,COX15,NDUFB2,PID1
ribonucleoside monophosphate biosynthetic process	GO:0009156	4	86	1,17E-04	6,76E-03	ATP5D,CHCHD10,IMPDH2,PID1

ribonucleoside triphosphate metabolic process	GO:0009199	6	270	1,34E-04	7,12E-03	ATP5D,CHCHD10,NDUFS7,COX15,NDUFB2,PID1
purine nucleoside triphosphate metabolic process	GO:0009144	6	271	1,37E-04	7,12E-03	ATP5D,CHCHD10,NDUFS7,COX15,NDUFB2,PID1
ribonucleoside metabolic process	GO:0009119	7	392	1,40E-04	7,12E-03	ATP5D,CHCHD10,NDUFS7,IMPDH2,COX15,NDUFB2,PID1
nucleoside monophosphate biosynthetic process	GO:0009124	4	94	1,65E-04	8,07E-03	ATP5D,CHCHD10,IMPDH2,PID1
nucleoside triphosphate metabolic process	GO:0009141	6	295	2,17E-04	9,91E-03	ATP5D,CHCHD10,NDUFS7,COX15,NDUFB2,PID1
purine nucleoside biosynthetic process	GO:0042451	4	102	2,26E-04	9,91E-03	ATP5D,CHCHD10,IMPDH2,PID1
purine ribonucleoside biosynthetic process	GO:0046129	4	102	2,26E-04	9,91E-03	ATP5D,CHCHD10,IMPDH2,PID1
ATP biosynthetic process	GO:0006754	3	48	3,74E-04	1,59E-02	ATP5D,CHCHD10,PID1
ribonucleoside biosynthetic process	GO:0042455	4	121	4,33E-04	1,78E-02	ATP5D,CHCHD10,IMPDH2,PID1
mitochondrial respiratory chain complex I assembly	GO:0032981	3	56	5,90E-04	2,21E-02	NDUFS7,ECSIT,NDUFB2
NADH dehydrogenase complex assembly	GO:0010257	3	56	5,90E-04	2,21E-02	NDUFS7,ECSIT,NDUFB2
mitochondrial respiratory chain complex I biogenesis	GO:0097031	3	56	5,90E-04	2,21E-02	NDUFS7,ECSIT,NDUFB2
purine ribonucleoside triphosphate biosynthetic process	GO:0009206	3	59	6,88E-04	2,50E-02	ATP5D,CHCHD10,PID1
purine nucleoside triphosphate biosynthetic process	GO:0009145	3	60	7,22E-04	2,55E-02	ATP5D,CHCHD10,PID1
ribonucleoside triphosphate biosynthetic process	GO:0009201	3	65	9,12E-04	3,11E-02	ATP5D,CHCHD10,PID1
purine ribonucleotide metabolic process	GO:0009150	7	537	9,28E-04	3,11E-02	ATP5D,CHCHD10,NDUFS7,IMPDH2,COX15,NDUFB2,PID1
generation of precursor metabolites and energy	GO:0006091	6	395	1,01E-03	3,30E-02	ATP5D,CHCHD10,NDUFS7,COX15,NDUFB2,PID1
ribonucleotide metabolic process	GO:0009259	7	552	1,09E-03	3,46E-02	ATP5D,CHCHD10,NDUFS7,IMPDH2,COX15,NDUFB2,PID1
purine nucleotide metabolic process	GO:0006163	7	556	1,14E-03	3,52E-02	ATP5D,CHCHD10,NDUFS7,IMPDH2,COX15,NDUFB2,PID1
ribose phosphate metabolic process	GO:0019693	7	567	1,27E-03	3,85E-02	ATP5D,CHCHD10,NDUFS7,IMPDH2,COX15,NDUFB2,PID1
mitochondrial respiratory chain complex assembly	GO:0033108	3	77	1,49E-03	4,28E-02	NDUFS7,ECSIT,NDUFB2
nucleoside triphosphate biosynthetic process	GO:0009142	3	77	1,49E-03	4,28E-02	ATP5D,CHCHD10,PID1
nicotinamide nucleotide biosynthetic process	GO:0019359	2	20	1,55E-03	4,28E-02	QPRT,ME1
pyridine nucleotide biosynthetic process	GO:0019363	2	20	1,55E-03	4,28E-02	QPRT,ME1
mitochondrion organization	GO:0007005	8	770	1,70E-03	4,60E-02	LRPPRC,CHCHD10,NDUFS7,MRPS15,LSM4,ECSIT,NDUFB2,PID1
mitochondrial ATP synthesis coupled electron transport	GO:0042775	3	82	1,79E-03	4,73E-02	NDUFS7,COX15,NDUFB2
purine-containing compound metabolic process	GO:0072521	7	605	1,84E-03	4,73E-02	ATP5D,CHCHD10,NDUFS7,IMPDH2,COX15,NDUFB2,PID1
organophosphate metabolic	GO:0019637	10	1167	1,86E-03	4,73E-02	ATP5D,CHCHD10,NDUFS7,IMPDH2,Q

process						PRT,COX15,DCTD,NDUFB,ME1,PID1
cofactor biosynthetic process	GO:0051188	4	181	1,94E-03	4,81E-02	QPRT,HMBS,COX15,ME1
ATP synthesis coupled electron transport	GO:0042773	3	85	1,98E-03	4,81E-02	NDUFS7,COX15,NDUFB2
organophosphate biosynthetic process	GO:0090407	7	614	2,00E-03	4,81E-02	ATP5D,CHCHD10,IMPDH2,QPRT, DCTD,ME1,PID1
heme biosynthetic process	GO:0006783	2	23	2,05E-03	4,82E-02	HMBS,COX15
organelle inner membrane	GO:0019866	8	564	1,69E-04	3,30E-02	ATP5D,LRPPRC,NDUFS7,MRPS15,COX15,ECSIT,NDUFB2,LGALS3

Results of GO Term enrichment analysis. The table lists GO term pathways (> 10 genes) involved in biological processes, the number of overlapping genes in the query gene list and each pathway, the size of each pathway, the nominal p value derived from a hypergeometric test and the corresponding FDR adjusted p-value.

Supplemental table 6 qPCR Primer sequences

Gene	Forward	Reverse
**36B4**	CGGGAAGGCTGTGGTGCTG	GTGAACACAAAGCCCACATTCC
**GUSB**	CACCAGGGACCATCCAATACC	GCAGTCCAGCGTAGTTGAAAAA
**B2M**	CCAGCAGAGAATGGAAAGTC	GATGCTGCTTACATGTCTCG
**ACBD6**	TTGGTGGGCCAGTTATTAGTTC	CCAGTGAAGTAGAGCCCTACC
**CHCHD10**	CTACCATGGTCTGAGCTCCC	CATTTGTGTCTTGGGTTATCTGTG
**CKS1B**	CGACGACGAGGAGTTTGAGTATC	TTAGGGACCAGCTTGGCTATGT
**COX15**	TGGAGTAACTAGGTTGACAGAG	TGGAAATTGCTGGTATCTTTGG
**ECSIT**	ACAGGAACCTCCATCTCTCAG	GGACTGTTCAGAGCTATGGGC
**FERMT3**	AGCAGATCAATCGCAAGCAG	ATCCCGTACTTGTCCAGTGT
**F13A1**	GTGAAGATGATGCTGTGTATCTG	ATGCCATCTTCAAACTGACC
**HMBS**	GGCAATGCGGCTGCAA	GGGTACCCACGCGAATCAC
**IFNA1**	GCCTCGCCCTTTGCTTTACT	CTGTGGGTCTCAGGGAGATCA
**IMPDH2**	GGGCATCATCTCCTCCAGGG	TGCTGCGCTGCAGAATTTCA
**LRPPRC**	GAGAGATGCCGGAATTGAGC	CTCGGACTTCTCCACCTTCT
**NME-1**	AAGGAGATCGGCTTGTGGTTT	CTGAGCACAGCTCGTGTAATC
**NME-2**	CATTGACCTGAAAGACCGAC	ATGATGTTCCTGCCAACCTG
**QPRT**	GGGCAGCCTTTCTTCGATG	GGAGCCCATACTTCTCCACCA
**TMEM134**	CAGTTCAGCATTGATGATGCC	TTCTCCAGGTTCTGGTAGCG
**USP49**	CTCATCCCCTTCTCCCAGAG	TTCCAGGGATAGGTCCCAAA
**ZWILCH**	TTGGCTGATGGTTTGAGGAC	TGGTATGAAATCACACTACTGCTC

Supplemental table 7 qPCR validation data

Gene	Fold Induction obese group (95% CI difference in fold induction)	P-value
**ACBD6**	0.59 (-0.18 to 1.0)	0.17
**CHCHD10**	1.13 (-0.63 to 0.37)	0.60
**CKS1B**	0.85 (-0.19 to 0.48)	0.38
**COX15**	1.13 (-0.97 to 0.72)	0.76
**ECSIT**	1.35 (-0.87 to 0.16)	0.17
**FERMT3**	1.04 (-0.44 to 0.36)	0.85
**F13A1**	1.28 (-0.88 to 0.32)	0.35
**HMBS**	0.62 (0.087 to 0.68)	0.01
**IFNA1**	1.23 (-1.04 to 0.59)	0.58
**IMPDH2**	0.64 (-0.014 to 0.74)	0.06
**LRPPRC**	0.60 (0.14 to 0.68)	0.005
**NME-1**	0.71 (-0.071 to 0.65)	0.11
**NME-2**	0.85 (-0.58 to 0.89)	0.68
**QPRT**	0.73 (-0.39 to 0.94)	0.41
**TMEM134**	0.32 (0.079 to 1.28)	0.028
**USP49**	0.84 (-0.28 to 0.60)	0.47
**ZWILCH**	0.45 (0.18 to 0.92)	0.005

Supplemental table 8 Clinical characteristics of the adult cohort at risk

**Variables**	
Age, years	62.4 (10.1)
Male sex	262 (74.6)
BMI, kg/m2	27.4 (4.3)
Obese (BMI>30)	80 (23.3)
Hypertension	229 (65.2)
Hypercholesterolemia	230 (65.5)
Diabetes mellitus	75 (21.4)
Current smoker	71 (20.2)
Positive family history	151 (43.0)
prior MI	111 (31.6)
prior PCI	130 (37.0)
prior CABG	32 (9.1)
SYNTAX score*	13 (6-22)

Baseline characteristics of the adults with cardiovascular risk (n=351). Discrete variables are given as absolute count (%), continuous variables as mean (SD) or as median (IQR). *median (IQR).

Supplemental figure 1 - Nodal map

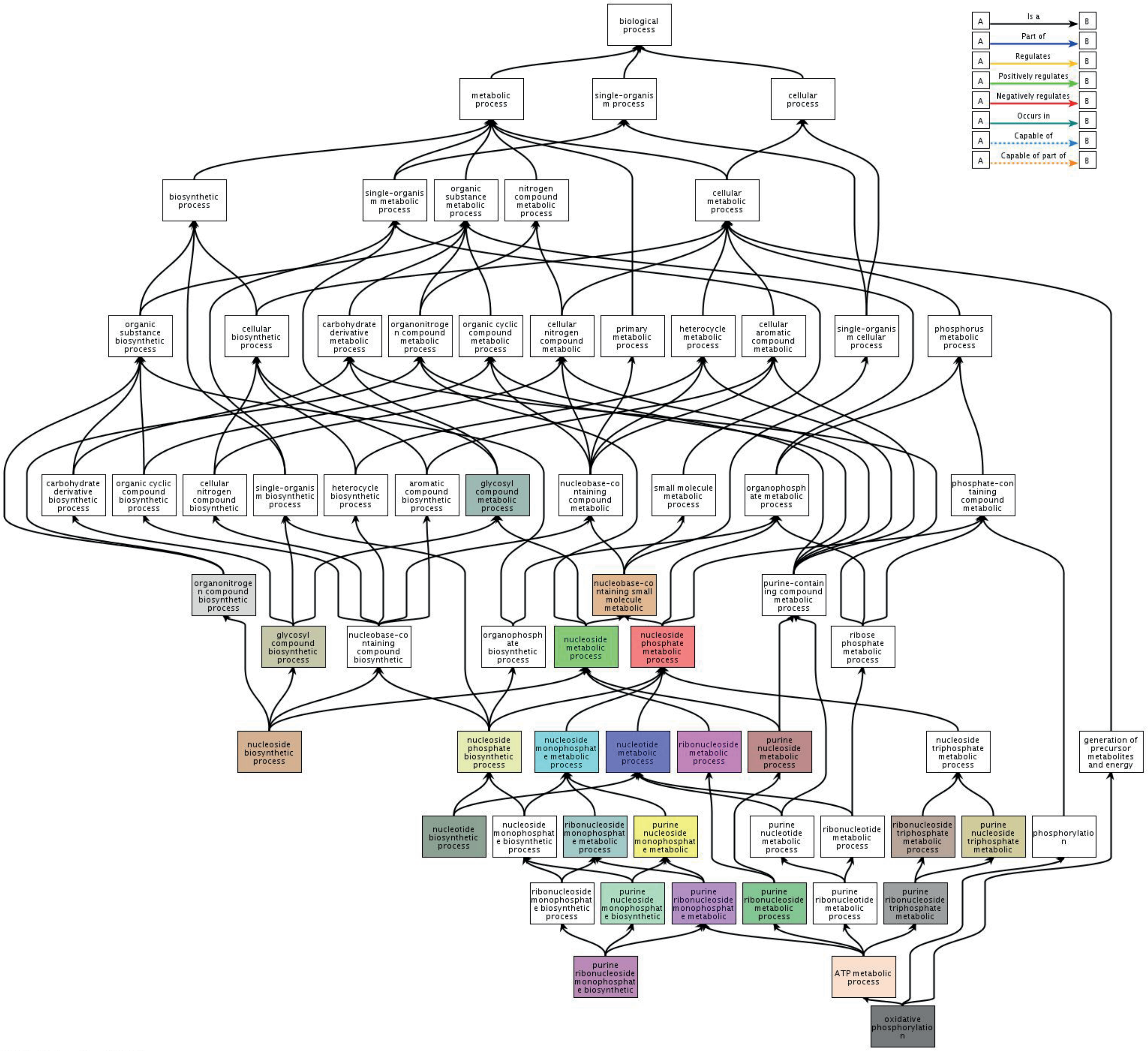

Supplemental figure 2 - Magnetic bead sorting

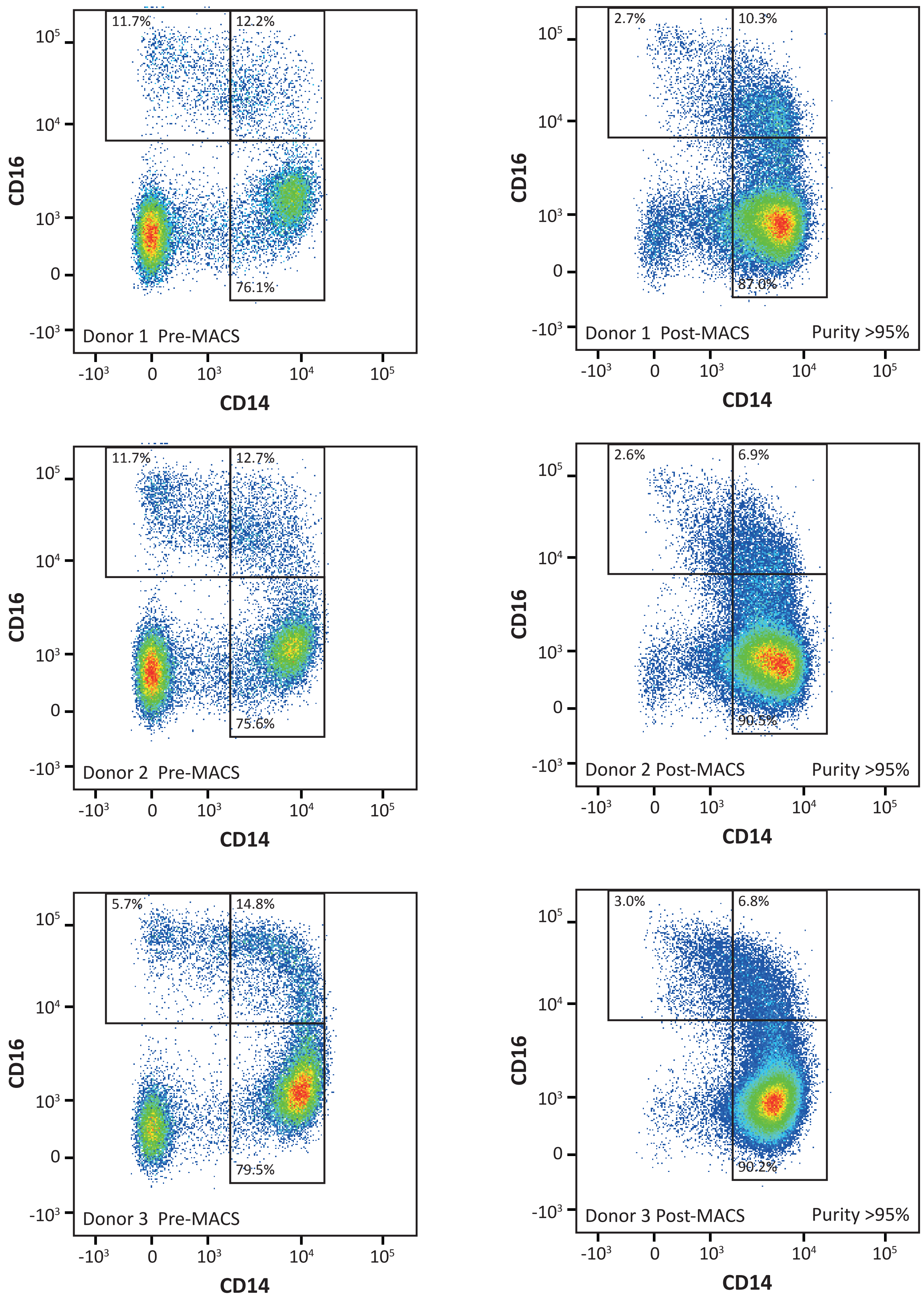

The monocyte subsets of 3 random patients before and after magnetic bead sorting are shown. Purity of the monocyte fraction after magnetic bead sorting was >95%.
